# Strategies to increase HIV testing among men who have sex with men and transgender women: an integrative review

**DOI:** 10.1186/s12879-023-08124-z

**Published:** 2023-04-18

**Authors:** Gustavo Machado Rocha, Raissa Carolina Fonseca Cândido, Nathália Pacífico de Carvalho, Emilly Gabrielly Araujo Carvalho, Alícia Amanda Moreira Costa, Ives Vieira Machado, Marcos Paulo da Cruz Pimenta, José Anastácio de Paula Júnior, Mark Drew Crosland Guimarães, Cristiane Aparecida Menezes de Pádua

**Affiliations:** 1 Federal University of São João del-Rei, Divinópolis, Brazil; 2 Department of Social Pharmacy, Federal University of Minas Gerais, Belo Horizonte, Brazil; 3 Department of Social and Preventive Medicine, Federal University of Minas Gerais, Belo Horizonte, Brazil

**Keywords:** Sexual and gender minorities, HIV seroprevalence, HIV testing, Point-of-care testing, Integrative review

## Abstract

**Background:**

Men who have sex with men (MSM) and transgender women (TGW) are disproportionately affected by HIV, with much higher incidence and prevalence rates than in the general population in different countries. There are several barriers to testing among MSM and TGW, such as low risk perception, anticipation of HIV-related stigma, discrimination of sexual orientation, in addition to difficulties related to care and access to health services. Therefore, analyzing the available evidence of the effectiveness of strategies for scaling up HIV testing among key populations is essential to point out potential knowledge gaps which may need to be addressed and develop public health policies to promote testing and early diagnosis of HIV infection.

**Methods:**

An integrative review was carried out to evaluate strategies for scaling up HIV testing in these populations. Search strategy was performed on eight electronic databases, without language restriction. We included clinical trials, quasi-experimental studies, and non-randomized studies. Study selection and data extraction were both performed independently by pairs and disagreements were solved by a third revisor. The screening of the studies was carried out through the selection of titles/abstracts and the reading of the full texts of the pre-selected studies based on the Preferred Reporting Items for Systematic Reviews and Meta-Analyses (PRISMA). Data extraction was performed using a structured form.

**Results:**

Thirty-seven publications referring to 35 studies were included, mostly being carried out in the United States of America and Australia. No studies were found evaluating disaggregated data on TGW. The studies were grouped into four types of intervention strategies: self-test distribution system (n = 10), organization of health services (n = 9), peer education (n = 6), and social marketing campaign (n = 10). Strategies that focused on the first three groups, combined or not, were more effective in increasing HIV testing among MSM.

**Conclusions:**

Considering the diversity of interventions and the methodological heterogeneity of the included studies, strategies especially involving self-test distribution systems, associated with new information and communication technologies, should be evaluated in different communities and social contexts. Research evaluating specific studies on TGW population is still needed.

## Introduction

HIV infection continues to represent a global health problem, with 1.5 million incident cases and 680,000 AIDS-related deaths in 2020 [1]. One of the great challenges in facing the HIV/AIDS epidemic, in line with the “95-95-95” target proposed by the Joint United Nations Program on HIV/AIDS (UNAIDS) [2], is to increase the capacity for identification of new cases for early diagnosis and immediate treatment, helping to control the spread of the virus [3, 4]. In 2020, it was estimated that 16% of people living with HIV were unaware of their HIV status [5]. Cisgender men who have sex with men (MSM), transgender women (TGW), and other key populations are disproportionately affected by HIV, with much higher prevalence rates than in the general population in different regions and countries. Worldwide, it is estimated that around 70% of new HIV infections occur in people from key populations or their sexual partners [5–8], and approximately one third of MSM and TGW are not aware of their HIV status [9, 10], with a low proportion of testing in the last 12 months worldwide [11–13]. Assessing more vulnerable populations, including MSM and TGW, for the provision of testing and treatment services is a key task in controlling the HIV epidemic.

Structural and individual factors have been identified as barriers to testing among MSM, such as low perception of their own risk of infection, fear of a positive result, anticipation of HIV-related stigma, discrimination, and acceptance of sexual orientation, in addition to difficulties and dissatisfaction related to health services [14–16]. TGW, commonly included in the MSM group in epidemiological studies, are poorly studied in relation to their particularities and vulnerabilities to HIV [17, 18]. Stigma and discrimination related to gender expression and identity and other negative experiences in health services can make it difficult for TGW to access HIV testing services [13, 18–21].

Strategies for scaling up HIV testing in these key populations are critical, including health education actions with peer involvement, strategies based on social networks, use of self-testing for HIV, distribution of testing kits outside health facilities, awareness campaigns in media and public spaces, in addition to changes of traditional care models [22, 23]. These strategies may be influenced by several contextual and structural factors, such as internet access, cultural adequacy, availability of public services, and human resources [22, 23]. Analyzing the available evidence of the effectiveness of these strategies in an integrated manner is essential to point out potential knowledge gaps which may need to be addressed and develop public health policies to promote testing and early diagnosis of HIV infection. Thus, the present integrative review was carried out with the objective of identifying strategies for the promotion of HIV testing in populations of MSM and TGW.

## Methods

### Study population

This is an integrative literature review of original articles published until September 2021, without restriction of publication date or language, evaluating strategies to promote HIV testing among the MSM and TGW populations.

### Types of study 

Clinical trials (randomized and non-randomized), quasi-experimental studies, and observational studies, including case–control, cohort, cross-sectional and ecological studies, which presented at least two measures of the primary event (e.g., before and after the intervention) or assessment in two different periods, were included.

Qualitative studies, case reports or series, letters to the editor, review studies, and those that included data from the study population only in aggregate form were not considered in the review.

### Types of HIV testing interventions

Studies that presented results of strategies implemented to promote HIV testing among MSM and/or TGW, whether or not compared with other strategies, were included. Strategies were classified into four main groups, according to the predominant characteristic of the intervention evaluated in the study: (1) self-testing delivery system; (2) organization of health services; (3) social marketing campaign; and (4) peer education.

### Primary outcome

The primary study outcome was increase in HIV testing after an intervention strategy. Different measures of occurrence of the primary outcome (e.g. absolute number of tests, proportion and frequency of testing) used by the authors were considered.

### Data collection and analysis

#### Electronic search

**Fig. 1:**
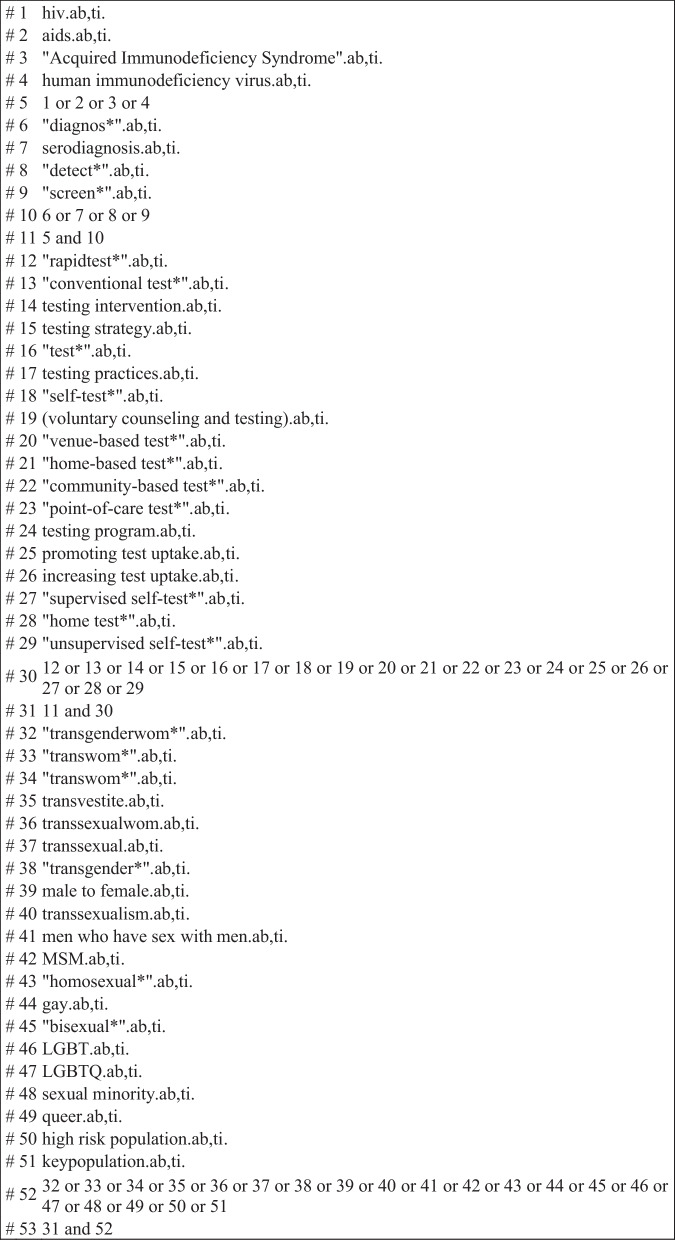
Strategy used to search for articles in the MEDLINE^TM^ (Medical Literature Analysis and Retrieval System Online) database via the Ovid^TM^ Platform

Searches were conducted in September 2021 in MEDLINE (via Ovid), Scopus, Sociological Abstracts (ProQuest), EMBASE, CINAHL, PsycINFO, Web of Science, and Global Index Medicus. The search strategy was carried out through a combination of free and indexed terms (Fig. 1), without restriction of publication date or language. Briefly, searches were performed using a combination of the following free and indexed terms and their respective synonyms, in addition to Boolean operators and truncation symbols: “HIV/aids; diagnosis/screening; testing; transgender; men who have sex with men”.

#### Selection of studies

The screening of records retrieved from electronic searches, after removing the duplicates, were performed in two steps, using the Rayyan^TM^ platform (https://www.rayyan.ai/) and Microsoft Excel^TM^ software. The first step consisted of selecting articles by reading titles and abstracts. In the second step, a complete reading of the pre-selected articles in the previous step was carried out.

In both steps, the selection of articles was carried out in pairs (total of nine researchers: GMR, RCFC, NPC, EGAC, AAMC, IVM, MPCP, JAPJ, CMP) independently. Disagreements were resolved by a third review (CMP, GMR). The selection process was summarized based on the PRISMA 2020 recommendation (Preferred Reporting Items for Systematic Reviews and Meta-Analyses) [24].

In the second step, the evaluation of the eligibility of the articles were ordered according to the following criteria: study design, intervention, comparison group, population, and outcomes, with the reasons for exclusion of studies duly documented and analyzed.

### Data extraction

Data from the included studies were extracted using a standardized form containing relevant information in the following domains: information on publication, study design, participants, evaluation, and results of primary and secondary outcomes. Data were extracted in pairs independently. Disagreements were resolved by consensus. The information extracted from all studies was checked by a third reviewer. Data were analyzed descriptively, and relevant results were reported narratively.

## Results

**Fig. 2:**
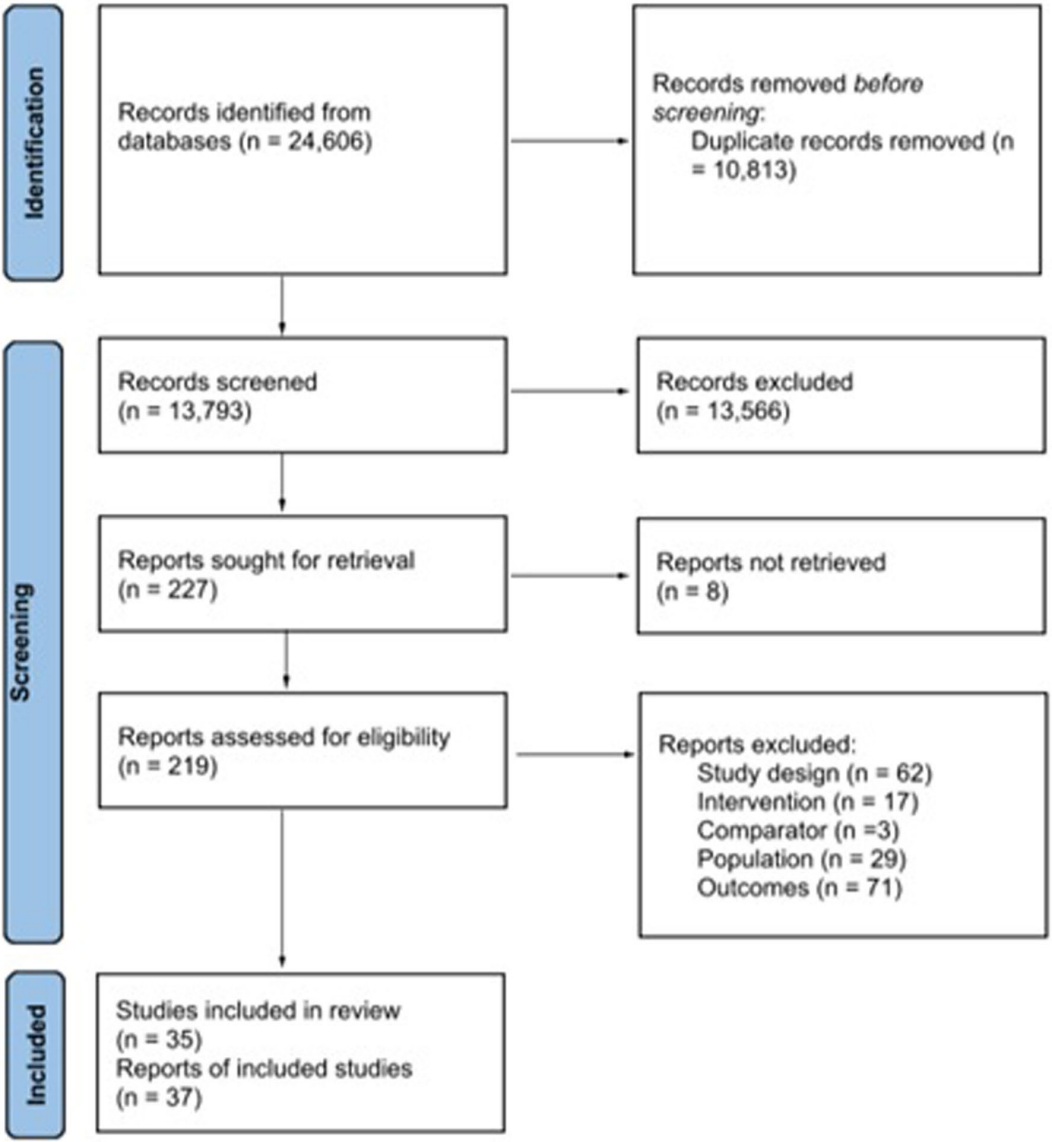
PRISMA diagram of studies searched and included in the review

In total, 24,606 records were identified, and 13,793 were evaluated after removing the duplicates. After evaluating the titles and abstracts, 227 publications were considered potentially relevant, and it was possible to access 219 (96.5%) for full reading and eligibility assessment. Thirty-seven publications referring to 35 studies met the selection criteria and were included in this review (Fig. 2). A total of 182 publications were excluded due to ineligibility regarding the study design (n = 62), the intervention (n = 17), the comparison group (n = 3), the population (n = 29) or the evaluated outcomes (n = 71).

The included studies (n = 35) were published between 2006 and 2021, eleven being conducted in Asia (seven in China, two in India, one in Taiwan and one in Vietnam), ten in the United States of America (USA), six in Australia and five in Europe (four in the United Kingdom—UK and one in Spain). Two studies were conducted in Africa (Tanzania and Nigeria), and only one study was conducted in Latin America (Peru). All included studies were written in the English language. Most of the studies consisted of controlled trials (n = 22; 19 of which were randomized and three were non-randomized), and there were also three quasi-experimental studies. Thirteen observational studies were included, three of which were prospective cohort studies, one retrospective cohort study, eight cross-sectional studies, and one ecological study.

The main characteristics of the studies are presented in Table 1, organized according to the type of strategy evaluated to increase HIV testing: self-test distribution system (n = 9), organization of health services (n = 10), social marketing campaign (n = 10), and peer education (n = 6). The sample size of most randomized clinical trials (n = 19) was less than 500 participants, with great variability according to the sample unit (individuals or number of tests performed). In observational studies, the number of participants also varied widely (Table 1).

**Table 1:** Synthesis of the characteristics of the included studies according to the type of intervention

Type of intervention	Author, year/location	Study design	N	Setting /Participants source	Strategy/intervention	Comparison groups	Outcome	Results
								HIV testing
Self-testing delivery system	MacGowan, 2020 [32] USA	RCT	2665	MSM frequented social media, music and dating websites	The distribution of HIV self-tests via mail to MSM recruited via the internet	Not receive HIV self-tests via mail	Frequency of HIV testing (mean number of times tested)	Mean number of tests over 12 months: Intervention: 5.3 / Control: 1.5 p < 0.001
	Zhang, 2020[35] China	RCT	216	Community and social media	Provision of free HIV self-testing (HIVST) kits + site-based HIV testing	Site-based HIV testing only	(i) HIV testing frequency (average number of HIV tests per participant); ii) HIV testing coverage (average number of HIV tests for sexual partners of MSM participants)	(i) HIV testing frequency Intervention: 3.75 / Control: 1.80 Standardized mean difference (SMD): 1.26 (95% CI 0.97–1.55), p < 0.001 Adjusted rate ratio: 2.10 (95% CI 1.75–2.53) (ii) HIV testing coverage Intervention: 2.65 / Control: 1.31 SMD 0.64 (95% CI 0.36–0.92), p < 0.001
	Rodger, 2020 [34] UK	RCT	10,111	Online platforms	Free delivery of free HIV self-testing kit to men recruited through online advertising	Control group did not receive free HIV self-testing kit	Proportion of HIV testing over 3 months follow-up	% tested Intervention: 97% / Control: 43% RR 2.27 (95% CI: 2.15–2.41)
	Zhu, 2019[31] China	RCT	100	MSM frequented community, commercial and online venues	Baseline procedures (video demonstration, questionnaire and self-test) + App (WeTest) usage to increase HIV testing through "oral fluid testing"	Only baseline procedures (video demonstration questionnaire and self-test)	Proportion of HIV testing	% tested Intervention: 92% / Control: 68% RR 1.99 (95% CI 1.03–3.84)
	Katz, 2018[26] USA	RCT	230	Sexual health center, social media and MSM frequented websites	Free access to HIV self-testing	Facility-based testing only	Frequency of HIV testing	Mean number of tests Intervention: 5.3 (95% CI 4.7–6.0) Control: 3.6 (95% CI 3.2–4.0) p < 0.0001
	Wang, 2018 [28] China	RCT	430	MSM frequented bars, clubs, saunas and websites	Promotion of HIV testing (4 min video) + motivational interview (MI) + home-based HIVST-OIC (online real-time instructions and pre-test/post-test counseling)	Promotion of HIV testing in general (3 min video)	Frequency of HIV testing within the 6-months follow-up	% tested Intervention: 89.8% / Control: 50.7% RR 1.77 (95% CI 1.54–2.03) p < 0.001
	Wray, 2018 [30] USA	RCT	65	MSM frequented dating apps, social media websites, and community	eTEST system: detects when users open the HIV self-test (HST) kits remotely in real time. Intervention groups: 1) eTEST—self-test with monitoring and 2) HBST—self-test without monitoring)	Control group received reminders to perform the test at clinics	Proportion of HIV testing	% tested eTEST: 100% HBST: 95% Control: 72% p < 0.001
	Jamil, 2017 [25] Australia	RCT	362	Sexual health centers; community-based organizations; social media and MSM frequented websites	HIV self-test + facility-based testing	Facility-based testing only	Number of HIV tests over 12 months	Number of tests Intervention: 701; tests per year: 4.0 (95% CI 3.7–4.3) Control: 313; tests per year: 1.9 (95% CI 1.7–2.2) RR 2.9 (95% CI 2.2–3.5)
	Vera, 2018 [27] UK	NRCT	232	MSM frequented sauna	Vending machine technology to distribute HIV self-test	Outreach testing by community workers	Number of HIV tests performed	Number of tests Intervention: 265 (34 tests per month) Control: 40 tests (6 tests per month)
	Banerjee, 2020 [33] UK	Retrospective cohort	1926	Sexual health centers	Home-based testing service available for online request as an alternative to attending a clinic of the “Umbrella” sexual health services	Clinic-based testing	Number of patients who requested home-based kits or who attended the clinic	Number of patients: 138 Home-based testing (7.2% of all MSM) 1788 Clinic-based testing (92.8% of all MSM)
Health services organization	Hu, 2021 [43] China	RCT	187	Voluntary counseling and testing (VCT) clinic	Assisted partner notification (self-tests kits for partner or notification by a community health worker)	Passive partner notification (by index cases themselves)	Proportion of index cases who successfully had any sexual partner accessing testing	% tested Intervention: 35% / Control: 17% p = 0.004
	Solomon, 2019 [39] India	RCT	9,997	Community	Integrated care centers (ICC) focused on MSM that provide integrated testing, prevention and treatment services	Conventional care services	Proportion of HIV testing	% tested Intervention: 33.9% (95% CI 32.8–35.2) Control: 25.2% (95% CI 23.3- 27.1) RR 1.77 (95% CI 1.30–2.41)
	Read, 2018 [38] Australia	RCT	422	Sexual health center	Access to HIV testing without seeing a clinician	Consultation with a clinician at every HIV test	Frequency of HIV testing over 12 months	Tests / person-years Intervention: 453 / 205.6 Control: 432 / 204.0 IRR: 1.04 (95% CI: 0.89–1.22)
	Galvan, 2006 [36] USA	NRCT	3645	MSM frequented bars	To offer the Bundle protocol (package of screening tests)	To offer only routine HIV test	HIV testing uptake	% tested: Intervention: 10.2% / Control: 8.9% RR 1.59 (95% CI: 0.82—3.10)
	Snow, 2013 [37] Australia	NRCT	14,423	Clinics specialized in gay men's health	HIV testing after hiring a nurse specializing in sexual health	HIV testing in a clinic that did not have a specialized nurse	Proportion of HIV testing	% tested Intervention: 57% / Control: 37% p = 0.026
	Maruyama, 2021 [44] Tanzania	Cross-sectional	18,462 (number of tests)	Community	Differentiated service delivery (DSD) models providing client-centered HIV services tailored to key-populations	First year (year 1) of the intervention and the last year (year 4)	Annual number of HIV tests	Number of tests 1745 (year 1) / 5081 (year 2) 6471 (year 3) / 5165 (year 4)
	Luo, 2020 [42] China	Cross-sectional	548	HIV counseling and testing clinics	a) Direct notification of partners for information provided by the Index Case (IAPN); b) Training and Provision of rapid self-test for the Index Case to take to the partner (HIVST); c) Guidance for the Index Case inform the partner to seek testing (PR)	Testing and counseling of couples (Index case was oriented to return with partner) (CHCT)	HIV testing uptake	Intervention: IAPN: 94.2% RR 3.9 (95% CI 1.6–9.3) HIVST: 93.8% RR 3.4 (95% CI 0.4–30.0) PR: 89.5% RR 1.8 (95% CI 0.9–3.6) Control: CHCT: 82.2%
	Patel, 2021 [41] Australia	Cross-sectional	43,560 (MSM attending testing services 2010–2018)	Clinical and community HIV testing services	Government HIV strategies that aimed to increase HIV testing, including testing targets, innovations in clinic procedures, expanded testing modalities, health promotion, and workforce development	–	% MSM attending testing services who had an HIV test in the last 12 months and annual testing frequency	2010 vs. 2018: 12-monthly HIV testing uptake: 83.9% vs. 95.1% (AAPC = 1.3%, 95% CI: 0.9–1.6, p < 0.001) Annual HIV-testing frequency: 1.4 vs. 2.7 (AAPC = 7.5%,95% CI: 6.1–9.0, P < 0.001)
	Fernandez-Lopez, 2020 [40] Spain	Cross-sectional	–	Community attending a network of VCT services operated mainly by NGO	Community-based voluntary counseling and testing services (CBVCT) + point of care tests (POCT)	–	Increase of the absolute number of MSM tested after introduction of POCT	1995–2006: 167 to 380 MSM tested annually 2007–2018: 1201 to 7988 MSM tested annually
Social marketing campaign	Patel, 2016 [50] India	RCT	244	MSM frequented dating websites; social media website	Internet-based avoidance/approach framed messages for HIV testing and consistent condom use based on the information motivation behavioral skills model	Follow-up period and one of the message frames	Proportion of HIV testing in the last 6 months	% tested Baseline vs. follow-up: 31.5% Baseline; 43.8% Follow-up (p = 0.04) At follow-up: 38% Approach; 50% Avoidance (p = 0.18)
	Bauermeister, 2015 [48] USA	RCT	104	LGBTQ pride celebrations and magazines; MSM frequented bars and clubs; websites	A web-based tailored content to promote HIV/STI testing (“Get Connected!”)	Did not receive personalized tailored content	HIV testing uptake after 30-day follow-up	% tested: Intervention: 32.4% Control: 22.2% Chi-square 1.18 (non-significant)
	Blas, 2010 [46] Peru	RCT	459	MSM frequented commercial and advocacy websites	Online HIV-testing motivational videos	Online standard public health text	Proportion of HIV testing	% tested: Non-gay identified group Intervention: 11.3%; Control: 0% (p = 0.001) Gay-identified group Intervention: 5.6%; Control: 7.7% RR 1.07 (95% CI 0.40–2.85)
	Chiou, 2021 [55] Taiwan	Prospective cohort	831	Public hospital website (control participants); MSM frequented dating apps, social media websites (intervention)	Social networking voluntary counseling and testing (VCT) model, recruiting MSM from social networking platforms and conducting web-based discussions on dating apps and Facebook. It included scheduling through social networking platforms and providing VCT at designated times and places	Traditional voluntary counseling and testing (VCT) model, recruiting MSM through a website and testing station at a gay village	% of MSM who completed VCT after some interaction with the website (control) or social networking platforms—web-based discussion (intervention)	Intervention % completion of VCT after web-based discussion: 20.7% (215/1038) Dating apps: 52.8% (158/299) Facebook: 43.8% (21/48) Line app: 60% (36/60) Control % completion of VCT after browsing the website: 8,56% (616/7200)
	Hickson, 2015 [49] UK	Prospective cohort	2,047	MSM frequented dating websites; HIV survey	HIV health promotion media interventions targeting MSM named “I Did It” and “Count Me In” Subgroup 1 completed month 7 (incl. ‘I Did It’ measures) Subgroup 3 also completed month 13 (incl. ‘Clever Dick’ and ‘Count Me In’ measures)	MSM not aware of campaigns	Rate of HIV testing on months 7 and 13 (number of tests/person-years)	Rate of HIV testing (number of tests/person-years): - Recognized, or had seen and read, advertisement Subgroup 1: 1.67 / Subgroup 3: 1.60 - Not seen advertisement Subgroup 1: 1.19 / Subgroup 3: 1.12 Subgroup 1: RR: 1.37 (1.03 to 1.81), p = 0.03 Subgroup 3: RR: 1.48 (1.06–2.06), p = 0.02
	Tang, 2018 [53] China	Closed cohort stepped wedge cluster RCT	1,381	MSM frequented social media app	Multimedia HIV testing campaign, an online HIV testing service, and local testing promotion campaigns	Baseline period, before cities received the intervention	Proportion of HIV testing over 3 months follow-up	% tested Study period: 62% Intervention/post-intervention periods: 53% The Proportion of individuals testing during the intervention periods within a city was 8.9% (95% CI 2.2–15.5) greater than during the control periods Intention-to-treat analysis: the probability of an individual HIV testing during the intervention periods was higher than in the control periods (RR = 1.43, 95% CI 1.19–1.73)
	Wang, 2019 [54] China	Cross-sectional	15,932 (number of tested individuals)	MSM frequented social media app	Social media–based HIV testing promotion campaign (Blued), with a one-time mass massage push in 2015 encouraging users to get tested for HIV followed by monthly electronic banner promotions. In 2016, an online HIV testing appointment platform was embedded in the application	No comparison group, testing was assessed over time	Frequency of HIV testing	Number of tests per year - Before campaign: 836 (2013) / 425 (2014) - During and after campaign: 3.336 (2015) / 6.330 (2016) / 7.315 (2017)
	Van Handel, 2014[47] USA	Cross-sectional	NA (number of tests as unit of analysis)	National HIV survey	National HIV Testing Day (NHTD) Campaign (1 week—June 24–30, 2010)	Control week 1 (January 7–13, 2010) Control week 2 (August 12–18, 2010)	Number of HIV testing events in a week	Number of tests: Intervention—NHTD week: 5.919 Control week 1: 4.124 / Control week 2: 4.066 p < 0.001
	Wilkinson, 2016 [51] Australia	Quasi-experimental	48,892 (number of tests)	Sexual health centers, general practices specialized in gay men's health	"Drama Downunder campaign", a social marketing campaign using digital advertising material and printed material to promote regular sexual health screenings and increase sexual health awareness	Pre-DDU implementation period	Trends in number of HIV testing (January 2007–December 2013)	Difference on linear regression inclination: 1.7 (95% CI − 1.6 to 5.1)
	Guy, 2009 [45] Australia	Ecological	4,988 (survey); 2,717 (number of tested individuals)	Primary care clinics, sexual health centre, national MSM survey	Social marketing campaign (“Check-It-Out”): posters, cards, radio ads, publications and banners aimed at the gay public, and strategies for MSM not linked to the community (posters in public transport, educational spaces, ads on radio and newspapers). A website with HIV and STI information was developed	No comparison groups, HIV testing uptake during and subsequent to the campaign was compared	Proportion of HIV testing in the last 12 months (survey); number of HIV tests before and after the campaign (sentinel surveillance)	Survey -% tested During the campaign (2005): 61.4% After the campaign (2006): 61.9% p = 0.34 Sentinel surveillance—number of HIV tests April 2004 and August 2005: 3,435 202 HIV tests per month (average) No statistically significant difference
Peer education	Rhodes, 2017[58] USA	RCT	304	MSM frequented bars and clubs; community colleges and events; commercial venues; social media websites; mass media	*HOLA engrupos*, a small group peer education behavioral HIV prevention intervention designed to increase condom use and HIV testing among Hispanic/Latino gay, bisexual, and other men who have sex with men	General health education comparison intervention	Proportion of past 6-month HIV testing reported at the 6-month post intervention follow-up assessment	% tested Baseline Intervention: 32.45% / Control: 31.58% 6-month follow-up Intervention: 80.26% / Control: 27.63% OR 13.84 (95% CI 7.56–25.33) p = 0.001
	Young, 2013[59] USA	RCT	122	MSM frequented restaurants, clubs, schools, and universities; social media and websites	Delivery of HIV prevention and testing with peer leaders via Facebook groups over 12 weeks	Peer-delivered information on general health over Facebook	HIV test kit return rate	% returned test Intervention: 15.8% Control: 3.6%
	Outlaw, 2010 [56] USA	RCT	188	Community (MSM and general) venues	Motivational interviewing with field outreach	Traditional field outreach	Proportion of HIV testing immediately after the session	% tested Intervention: 49% /Control: 20% p = 0.001
	Rhodes, 2011[57] USA	Quasi-experimental	509	MSM frequented dating and social website	Cyber-Based Education and Referral/testing, an online peer education intervention based on social cognitive theory, empowerment education and natural helping	No comparison group, testing was assessed before and after the intervention	Proportion of HIV testing in the past 12 months	% tested Pretest: 44.5% / Posttest: 59.4% OR 1.8 (95% CI 1.4–2.5) p < 0.001
	Sabin, 2019 [61] Vietnam	Cross-sectional	407	Community	The President’s Emergency Plan for AIDS Relief (PEPFAR), a community outreach program based on peer education, employing outreach workers to expand community based HIV prevention measures among key populations	MSM without recent exposure to the outreach programs	Proportion of HIV testing	% tested Intervention: 67.7% /Control: 34.7% p < 0.001
	Adebajo, 2015 [60] Nigeria	Cross-sectional	6265	HIV counseling and testing program	3 HIV counseling and testing (HCT) intervention strategies: S1 (standard mobile outreach service); S2 (integrated mobile outreach service); S3 (peer-based outreach service)	A comparison was made between the three intervention strategies described	Uptake of HCT between the three strategies	Strategy 1 vs. 2: OR 0.29 (95% CI 0.22–0.40) p < 0.0001 Strategy 1 vs. 3: OR 9.21 (95% CI 5.57–15.23) p < 0.0001

LGBTQ: lesbians, gays, bisexuals, transsexuals, queer and other gender and sexuality groups; MSM: Men who have sex with men; NGO: Non-governmental organizations; NRCT: Non-randomized controlled trial; RCT: Randomized controlled trial; VCT: Voluntary counseling and testing; AAPC: annual average percentage changes

All studies included only MSM as a target population, and we were not able to retrieve any study on TGW using the specified research strategy. In 15 studies, most participants were aged between 16 and 30 years old, while in twelve, the predominant group was older (> 30 years old). Information on sexual orientation was described in 18 studies, in which the majority of participants identified themselves as gay or homosexual (a proportion ranging from 58 to 94%). Among the included studies, 19 (54.3%) presented the results of positivity for HIV in the studied sample, which ranged from 0.23% to 15.50%, depending on the design, the inclusion criteria, and other methodological strategies adopted in each study.

### Self-test distribution system

Eleven publications [25–35] from ten different studies (eight randomized controlled trials, one non-randomized clinical trial, and one retrospective cohort study) that evaluated different strategies for distributing and promoting self-testing in the MSM population were identified.

Zhu et al. [31] conducted a randomized clinical trial in China with MSM recruited from the community, commercial establishments, and on virtual social networks (68% aged between 18 and 29 years old). All study participants received two HIV self-test kits, and those in the intervention group additionally received access to a virtual platform (WeChat) via a cell phone application (WeTestApp) where they had access to videos, messages, and specific information about HIV prevention and testing. The proportion of testing was significantly higher in the intervention group compared to the control (92% vs. 68%; p = 0.004; Relative risk; 95% confidence interval—RR = 1.99; 95% CI = 1.03–3.84).

In another randomized clinical trial [30] involving MSM from the USA (mean age 35.5 years), participants in the intervention group received a kit to perform HIV self-testing at home, with the possibility of accessing a cell phone application to monitor testing, while those in the control group received a letter to get tested in a local clinic. In the intervention group, the proportion of testing was 100%, compared to 72% in the control group (p < 0.001).

MacGowan et al. [32] conducted a randomized clinical trial in the USA including MSM recruited from virtual platforms (social media, music and dating pages), where all participants had access to a database on remote testing and counseling by telephone. In the 12-month follow-up, the frequency of testing was higher among participants who received self-tests by regular mail (intervention group) than among those who underwent conventional face-to-face testing (control group) (mean of 5.3 and 1.5 tests, respectively; p < 0.001).

Another randomized clinical trial conducted in the USA [26] evaluated the effectiveness of the self-test strategy compared to usual testing among MSM (median age 35.5 years) at high risk of HIV infection recruited from sexual health clinics, social media, and web pages used by MSM. After a follow-up period of approximately 15 months, the mean number of tests was 5.3 in the intervention group and 3.6 in the control group (p < 0.0001).

In a non-randomized clinical trial conducted in the UK (37.0% aged between 45 and 64 years old), Vera et al. [27] evaluated the effectiveness of the availability of automatic vending machines for the sale of self-test kits on the frequency of testing among MSM users of saunas between September and December 2015. The intervention group (users of saunas with vending machines) performed 265 self-tests over the course of the trial (equivalent to 34 tests per month), while the control group (users of saunas without vending machines) underwent 40 tests performed by community professionals over the course of the trial (equivalent to 6 tests per month).

In the randomized clinical trial conducted by Jamil et al. [25] in Australia, participants (mean age 35 years) recruited from sexual health clinics, community organizations, social media, and web pages were allocated to the intervention group (free self-test kits and facility-based testing) or to the control group (conventional facility-based testing only) to assess the number of tests performed over 12 months of follow-up. Testing frequency was higher in the intervention group (701 tests; mean of 4.0 tests per year) than in the control group (313 tests; mean of 1.9 tests per year; p < 0.0001).

A randomized clinical trial conducted in China [28] evaluated three combined intervention strategies (online social marketing campaign, self-test delivery system with pre- and post-test guidance, and motivational interviewing) in the proportion of testing among 430 MSM during 6 months of follow-up. Participants were recruited from saunas, bars, and specific websites, with a predominance of age between 18 and 30 years old. In the group undergoing the intervention, 89.8% were tested for HIV, compared to 50.7% of the control group, which received only an online social marketing campaign (RR = 1.77, 95% CI = 1.54–2.03; p < 0.001).

A retrospective cohort study carried out in the UK [33] showed that between July and December 2017, fewer MSM used a system for dispensing HIV home testing kits via online ordering (n = 138), when compared to usual testing in health services (n = 1788). In contrast, a randomized clinical trial conducted by Rodger et al. [34] in the UK among MSM recruited in digital media found a significantly higher proportion of testing in the group that received HIV self-test kits, when compared with the control group (97% vs. 43%; p < 0.001). Similarly, a randomized clinical trial conducted in China [35] evaluated the effect of distributing HIV self-test kits compared to site-based testing on testing frequency over 12 months of follow-up. Higher frequency of testing was found among participants and their sexual partners in the intervention group (mean of 3.75 tests per participant and 2.65 tests per partner in the intervention group, compared to 1.80 per participant and 1.31 per partner in the control group; p < 0.001).

### Organization of health services

Nine studies that evaluated strategies for scaling up HIV testing focused on the organization of health services (three randomized clinical trials, two non-randomized clinical trials, and four cross-sectional studies) [36–44].

In the study conducted by Read et al. [38] in a period of 12 months in Australia, no difference was observed in the frequency of testing between the intervention (HIV testing without going through a medical consultation) and control groups (medical consultation at each HIV test performed) (453 tests per 205.6 person-years vs. 432 tests per 204.0 person-years; RR = 1.04; 95% CI = 0.89–1.22; p = 0.63). In contrast, Snow et al. [37] observed an increase in the proportion of testing in a clinic after hiring a nursing professional who specialized in sexual health (57% vs. 37%; p = 0.026). Solomon et al. [39] observed a higher proportion of testing over 24 months in clinic-based services with integrated prevention, testing, and treatment actions focused on MSM, compared to services with conventional organization of care (with non-integrated actions) in India (33.9% vs. 25.2%; RR = 1.77; 95% CI = 1.30–2.41).

A cross-sectional study conducted in 23 testing and counseling centers in two municipalities in China [42] evaluated four different strategies for tracing sexual contacts of newly HIV-diagnosed MSM: (1) couples’ HIV counselling and testing (CHCT), when the index case was requested to return to the clinic with his sexual partner at a subsequent date to receive joint HIV testing and counseling; (2) information assisted partner notification (IAPN), with direct notification of the exposed partner by the health team; (3) assisted HIV self-testing (HIVST), when the exposed partner performed self-test after index case training; and, (4) patient referral (PR), with only reference and guidance from the partner exposed by the index case. Higher proportions of testing were observed in the IAPN, HIVST and PR groups (94.2%, 93.8% and 89.5%, respectively), compared to the CHCT group (82.2%). The increase in testing was significantly greater in the IAPN group, whose partners were notified directly by the healthcare team (RR = 3.9; 95% CI = 1.6–9.3).

A randomized clinical trial carried out in China [43] found a higher proportion of HIV testing among sexual partners of newly diagnosed HIV index cases who received an assisted reporting strategy (dispensing of self-test kits for the index case to pass on to the partner or notification by trained health professionals from community organizations), compared to those who received passive notification directly from the index case themselves (34% vs. 17%, respectively; p = 0.004).

A non-randomized clinical trial [36] evaluated whether offering HIV testing along with screening tests for a group of other conditions would increase HIV testing among Hispanic or Latino MSM who frequented gay bars in USA. The proportion of testing was slightly higher in the intervention group (10.2% vs. 8.9%), without showing statistically significant difference (RR = 1.59; 95% CI = 0.82–3.10).

Fernandez-Lopez et al. [40] evaluated the trend of HIV testing by MSM in a time series in Spain, and found an increased number of tests performed annually after the introduction of rapid testing in community testing services (from 167 to 380 annual exams between the years 1995 and 2006, to 1201 to 7988 annual exams between the years 2007 and 2018). Patel et al. [41] evaluated the potential effect of multiple government strategies on HIV testing coverage, including innovations in care routines, human resources development in health, expansion of testing modalities, and health promotion campaigns, in the period from 2010 to 2018 in Australia. Overall, the results showed a significant increase in the uptake of HIV testing among MSM who attended testing services (from 83.9% in 2010 to 95.1% in 2018; p < 0.001) and the average frequency of HIV testing (from 1.4 to 2.7 annual tests; p < 0.001). Similarly, a cross-sectional study carried out in Tanzania [44] evaluated the effect of a strategy based on a differentiated service delivery model, where health teams traveled to different regions of the country, in locations where the MSM community lived, worked or socialized, to promote HIV testing and counseling. The study showed a significant increase in the annual number of tests among the MSM population, from 1745 in the first year to 5081 in the second year, 6471 in the third year and 5165 in the fourth year of evaluation after the implementation of the strategy.

### Social marketing campaign

Eleven articles from ten different studies evaluated the effect of social marketing campaigns on HIV testing [45–55], three of which were randomized clinical trials (USA, India, and Peru), two quasi-experimental studies (China and Australia), two cross-sectional studies (China and USA), two prospective cohort studies (UK and Taiwan), and one ecological study (Australia).

Five articles out of four studies conducted in Australia, China, and the United States [45, 47, 51–53], evaluated the effect of advertising campaigns, carried out both on virtual platforms and in person in the community, on the proportion or number of tests for HIV carried out in the respective localities. The interventions used printed educational material, audios, videos, dating mobile phone apps and specific internet pages aimed at the MSM population, with information on sexual health, HIV prevention, and testing. Wilkinson et al. [51] observed a progressive increase in the number of HIV tests over time in Australia, although there was a modest and non-significant increase in testing before and after the intervention (difference in the slope of the linear regression line of 1.7; 95% CI = 1.6–5.1). Tang et al. [52, 53] evaluated the increase in testing before and after carrying out multimedia campaigns associated with an online testing service and local testing promotion campaigns in eight cities in China. After 12 months of observation, the study reported that 62% of subjects underwent testing during the study period, with 53% during and after the intervention. Results from the intent-to-treat analysis showed that the probability of an individual testing for HIV during intervention periods (including post-intervention periods) was significantly higher than during control periods (RR = 1.43; 95% CI = 1.19–1.73; p < 0.001). Van Handel et al. [47] evaluated the effect of annual national campaigns on increasing HIV testing in the USA. The authors showed that the number of tests performed by the MSM population was significantly higher (p < 0.001) in the week of the educational campaign (n = 5919) when compared to the other weeks without intervention (n = 4124 and n = 4066). In contrast, Guy et al. [45] found similar proportions of testing before and after a specific advertising campaign (60.3% and 61.4%, respectively; p = 0.34) in sentinel clinics in Australia.

Among the included studies, six carried out advertising campaigns exclusively on virtual platforms as a strategy to increase HIV testing, with three randomized clinical trials (conducted in India, Peru, and the USA), two concurrent cohort studies (UK and Taiwan) and one cross-sectional study (China). In general, these studies used educational material (e.g., text messages and/or videos) in specific applications or websites aimed at the MSM population. Among clinical trials, Patel et al. [50] showed a slight increase in the proportion of testing in the intervention group after 3 months (31.5% vs. 41.8%; p = 0.04). Blas et al. [46] demonstrated some effectiveness in expanding HIV testing in the population that did not identify as “gay” (testing proportion of 11.8% in the group that received the intervention and zero in the control group; p = 0.001). In the study conducted by Bauermeister et al. [48], there was no significant difference in the proportion of testing between the two groups (31.5% vs. 25.9%; p = 0.27).

In the cohort study carried out by Hickson et al. [49], the effect of different educational health promotion strategies on the frequency of HIV testing among users of social networking sites was evaluated at three moments during the 13-month study. After seven months, the average annual number of tests per person was 1.67 among the exposed and 1.19 among the unexposed (RR = 1.37; 95% CI = 1.08–1.81; p = 0.03). After 13 months, the average annual number of tests per person was 1.60 among the exposed and 1.12 among the unexposed (RR = 1.48; 95% CI = 1.06–2.06; p = 0.02). In a prospective cohort study, Chiou et al. [55] found a higher proportion of testing among high-risk MSM in the intervention group (in which participants were recruited by social network platforms and tests were performed at a designated place by the participants) than in the control group (in which participants were recruited by a public website and tests were performed on traditional outreach screening station) (20.7% vs. 8.6%, respectively). Finally, the observational (panel-type) study conducted in China [54] showed a significant increase in the number of tests among the MSM population in the two years after a specific advertising campaign (carried out between 2015 and 2017), when compared to two previous years (836, 425, 3336, 6330 and 7315 tests were performed for HIV respectively in the years 2013, 2014, 2015, 2016 and 2017; p = 0.007).

### Peer education

Six studies used peer education strategy to increase HIV testing [56–61], two with a cross-sectional design (Nigeria and Vietnam), a quasi-experimental study (USA), and three randomized clinical trials (USA). The average age of participants ranged from 19 to 37 years.

Rhodes et al. [58] evaluated the proportion of testing between the intervention (peer education in small groups, based on social cognitive theory, empowerment education, and traditional Hispanic/Latino cultural values) and control (general health education) groups over a six-month period in North Carolina, USA, and identified a significant increase in the proportion of HIV testing in the group that underwent the intervention (80.3%) compared to the control group (27.6%) (RR = 14.9; 95% CI = 7.56–25.33; p < 0.001).

Sabin et al. [61] evaluated the overall proportion of HIV testing after implementation of a community-based outreach program based on peer education in a community in Vietnam, identifying a significant increase in the overall proportion of testing after the start of the program (34.7% at the beginning of the program; 67.7% after using the strategy; p < 0.001).

Adebajo et al. [60] evaluated three distinct educational strategies carried out by peers for testing in decentralized community services in Nigeria (standard testing service, integrated mobile testing service, and peer testing service). HIV testing was significantly higher in the group that received the peer testing service when compared to the standard mobile testing service group (RR = 9.21; 95% CI = 5.57–15.23; p < 0.001).

In the randomized clinical trial conducted by Outlaw et al. [56] in the USA, researchers evaluated the effect of motivational interviewing delivered by trained peer outreach workers on testing among young MSM (mean age 19.8 years). The testing proportion was higher in the intervention group (49%) compared to the group that received only peer education actions (20%; p = 0.001).

Two studies, both conducted in the USA, adopted the peer education strategy exclusively on virtual platforms [57, 59] with a mean age between 30 and 40 years old. Young et al. [59] performed a randomized clinical trial that showed a higher proportion of HIV home self-test return among those exposed to peer-reported information related to HIV prevention and testing (15.8%), compared with those who received only general health information in online discussion groups (3.6%). Rhodes et al. [57] evaluated the proportion of testing in the last 12 months after the introduction of an educational intervention (based on social cognitive theory, empowerment education, and natural help) with guidelines related to HIV prevention carried out by peers on online relationship and message exchange platforms (chat). The proportion of HIV testing increased from 44.5% to 59.4% before and after the intervention, respectively (RR = 1.8; 95% CI = 1.4–2.5; p < 0.001).

## Discussion

This study presents findings from a comprehensive integrative review to identify HIV testing promotion strategies targeted at MSM and TGW. The studies included in this review adopted strategies focused on self-test delivery systems, organization of health services, social marketing campaigns, or peer education. According to the results analyzed, each type of intervention has advantages and disadvantages, with potential specific recommendations for new actions and scientific research (Table 2).

**Table 2:** General summary of characteristics and recommendations by type of intervention

Intervention group	Advantages	Disadvantages	Recommendations	Gaps for new research
Self-test distribution system	Use of new technologies for health promotion, aimed at different contexts; possibility of approaching subgroups that are more difficult to access; more controlled clinical trials with more robust evidence of efficacy	Higher operational cost; greater difficulty in carrying out post-test counseling and in offering comprehensive care for new cases; difficulty to reach populations of greater social vulnerability	Application development for specific local contexts; incorporation of self-test distribution strategies into the routine of Testing and Counseling Centers (CTA)	Clinical trials with robust samples and longer duration; assessment in places of greater socioeconomic vulnerability
Organization of health services	Relatively simple execution; possibility of offering counseling and comprehensive care for new cases; assistance by a qualified professional with the potential to promote testing	Does not reach subgroups that are more hidden and resistant to testing; cost of hiring and permanent training of human resources	Creation of specific services for MSM and sexual health; permanent qualification of CTA teams; employment of actions for the active search for sexual contacts	Clinical trials with robust samples and for a longer period of time; cost-effectiveness assessment of actions
Social marketing campaign	Relatively simple execution; greater reach in the general community; possibility of approaching subgroups that are more difficult to access through social media actions	Efficacy in general with more discrete magnitude and decreasing with time; difficulty in reaching populations with greater social vulnerability; need to carry out frequent campaigns associated with other actions	Expansion of educational actions on social networks and other virtual platforms, especially those aimed at specific subgroups of key populations	Clinical trials using different digital resources, with robust and longer-lasting samples; employing the most appropriate means of communication to reach the target population
Peer education	Content of actions adapted to the context of the target population; possibility of approaching subgroups that are more difficult to access (especially in virtual environments)	More difficult to operationalize, especially actions in the field aimed at subgroups that are more difficult to access; higher cost for training the team	Development of strategies to encourage the involvement of civil society and Non-Governmental Organizations in testing and prevention actions	Randomized clinical trials with evaluation of a greater number of participants and for a longer period, including a diversified spectrum of virtual environments

Considering the knowledge gap on testing strategies aimed at TGW and the inadequacy of evaluating them together with the MSM group, it was decided to restrict the selection to exclusive studies involving the trans population. However, by the established search criteria, no studies with disaggregated data on TGW were identified, which points to the need for efforts to develop specific studies to reach and promote testing by the population of transgender women. The population of TGW has unique gender identities, social and behavioral vulnerabilities, such as stigma and discrimination, lack of social and legal recognition of their affirmed gender, and exclusion from employment and educational opportunities, leading to disproportionately low rates of HIV testing and high rates of HIV infection. Therefore, it is necessary to expand the field of HIV research and prevention actions specifically among trans population, avoid conflation of TGW with MSM in these strategies and addressing these trans-specific issues. [17–19]

Free of charge self-test distribution has been shown to be effective in promoting testing among MSM when combined [25, 27, 28, 30, 31, 35] or not [26, 32, 34] with other strategies. HIV self-test distribution strategy showed to be useful for high-risk and infrequent MSM testers, which has been thought to reduce operational barriers of clinic-based testing and stigma and discrimination [25, 26], as well for reaching MSM partners [35] and members of social network [32]. This is in line with what have been described that MSM have strong social networks [7].

By comparing HIV self-testing distribution to clinic-based testing, an important issue that emerged from the review is whether the self-testing strategy would complement [25, 35] or replace [26] the clinic-based testing, pointing out the potential for obtaining poor outcomes concerning sexually transmitted diseases testing and HIV transmission as well by the replacement with self-tests. The type and quality of the available products will play an important role in early HIV detection, acceptability, and willingness to test [25, 26, 31, 62–64]. Another concern regarding the strategy refers to the confirmation of positive results of self-tests, the occurrence of psychological harms in the absence of post-test counseling and the referral of MSM to health care [25, 27, 28, 32, 35]. It is possible to deal with these issues using communication technologies with remote face-to-face interaction, which can be a limiting factor due to the higher costs and difficulty of access [28, 30, 31].

Organization of health services included heterogeneous strategies organized and operated by the health services, addressing the MSM population in communities [36, 39, 44], in sexual health clinics, and testing and counseling clinics [37, 38, 42, 43] or in both clinical and community settings [40, 41]. In general, the provision of health services with integrated prevention, testing, and treatment actions provided by a trained multidisciplinary team can be effective in promoting testing, although it is necessary to expand access and use of these services by the MSM population, specially by subgroups that are more hidden and resistant to use health care facilities [37–39]. Moreover, well training professionals can increase testing rates for HIV and other STIs among MSM and partners of newly HIV-diagnosed patients, although this may incur higher costs [37, 38, 42, 43].

Strategies using social marketing campaigns resulted in an increase in the proportion of testing in two [46, 50] of the three randomized controlled trials evaluated. In general, these strategies should be performed periodically, as their efficacy tends to be more discrete and to decrease with time [45, 51], combining interventions on geosocial networking platforms and HIV testing service announcements [65], and including different social actors in its construction (e.g., members of key populations, lay people, and experts) [52]. Messages based on a gain–loss framework can be used, although the loss approach is indicated as more appropriate for the diagnosis (e.g., diagnostic tests), while the gain approach is better suited to prevention measures (e.g., condom use) [66]. These results reinforce the need to expand studies and propose new strategies that assess structural changes and other public policies in conjunction with health promotion actions to obtain better testing indicators [67].

Peer education has shown to be a potential strategy to increase HIV testing in the MSM population, despite the studies being heterogeneous in terms of methodology. In contrast, the evidence found for peer education on virtual platforms is fragile, considering the methodological limitations of quasi-experimental studies [57], due to the absence of randomization and a comparator group, in addition to sample size limitations [59]. It is noteworthy however, that most of the studies were published until 2015, a period in which the profile of use of digital social networks was different and interventions through these communication channels were less frequent.

## Conclusion

The evidence of efficacy and effectiveness of strategies for increasing testing among MSM emerged from observation studies and controlled trials conducted in different settings. The findings cannot be directly extrapolated to other MSM populations due to cultural, social and economic specificities, in addition to methodological aspects of the studies that prevent generalization. Nevertheless, this integrative review provided a synthesis of the main strategies assessed worldwide and should contribute to the adoption of strategies in similar contexts. Research evaluating specific studies on TGW population is still needed.

Considering the diversity of proposed interventions and the methodological heterogeneity of the studies included in this review, it is recommended to evaluate strategies especially involving self-test distribution systems, associated with new information and communication technologies, applied in different communities and social contexts.

## Data Availability

The datasets used and/or analysed during the current study are available from the corresponding author on reasonable request.

## Abbreviations

AIDS: Acquired immunodeficiency syndrome
CHCT: Couples’ HIV counselling and testing
CI: Confidence interval
HIV: Human immunodeficiency virus
HIVST: HIV self-testing
IAPN: Information assisted partner notification
MSM: Men who have sex with men
PR: Patient referral
PRISMA: Preferred Reporting Items for Systematic Reviews and Meta-Analyses
RR: Relative risk
TGW: Transgender women
UK: United Kingdom
UNAIDS: Joint United Nations Program on HIV/AIDS
USA: United States of America
